# Erratum: An enterovirus strain isolated from diabetic child belongs to a genetic subcluster of echovirus 11, but is also neutralised with monotypic antisera to coxsackievirus A9

**DOI:** 10.1099/jgv.0.002164

**Published:** 2025-10-16

**Authors:** Haider Al-Hello, Anja Paananen, Mervi Eskelinen, Petri Ylipaasto, Tapani Hovi, K. Salmela, Alexander N. Lukashev, Shubhada Bopegamage, Merja Roivainen

**Affiliations:** 1 Enterovirus Laboratory, National Public Health Institute, Helsinki, Finland; 2 Renal Transplant Unit, Helsinki University Hospital, Helsinki, Finland; 3 Institute of Poliomyelitis and Viral Encephalitis, Russian Academy of Medical Sciences, Moscow, Russia; 4 Department of Virology, Slovak Medical University, Bratislava, Slovakia

The eighth author’s name was spelled incorrectly in the published version of this article.

Previously, it was spelled as Shubhada Bobegamage. It should have been rightly spelled as Shubhada Bopegamage.

The Microbiology Society apologises for any inconvenience caused. The online version of the original article has been updated to reflect this correction.

